# Supplementary material for: Super-resolution imaging for the detection of low-energy ion tracks in fine-grained nuclear emulsions
Source: Sci Rep. 2023 Dec 20;13:22813. doi: 10.1038/s41598-023-50208-y (PMC10739808; doi:10.1038/s41598-023-50208-y)
Supplement: Supplementary file 1 — Supplementary Information. [file 41598_2023_50208_MOESM1_ESM.pdf]

The events matching procedure.

Supplementary figure 1 and Supplementary figure 2 show overlayed images taken at the SEM and the optical microscope. The SEM images, highlited red, were 3×-downscaled and rotated manually to match approximately the optical microscope images. The latter ones were composed of individual event images since the DAQ software controlling the optical microscopes, for the sake of the memory optimisation, does not save the whole image but only 80×80 pixels around each detected event. Nevertheless, pixel offsets of event images were also saved making it possible to reconstruct their original position inside the field of view. The missing pixels containing no signal are set black.

Both the pattern matching and cropping around events in SEM images were performed manually ensuring that the event is present in both images at the same place inside the corresponding pattern. The yellow arrow in Supplementary figure 2 indicates the position of the event shown in Figure 7 of the article.

Original SEM and optical microscope images, as well as the overlayed ones are available in supplementary materials. All 104 events analysed in the article are contained in these two fields of view.

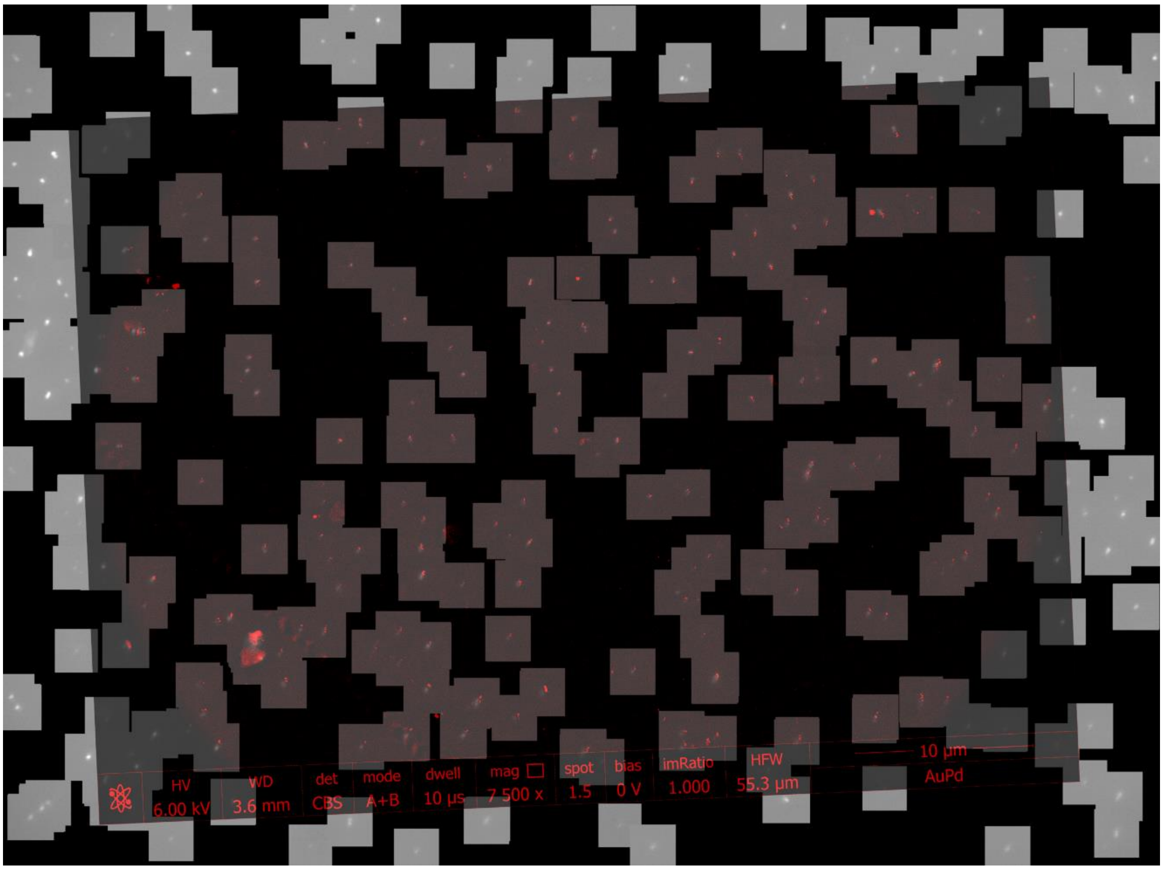

Supplementary figure 1. Overlayed SEM and optical microscope images for the first view. The SEM image is highlighted red.

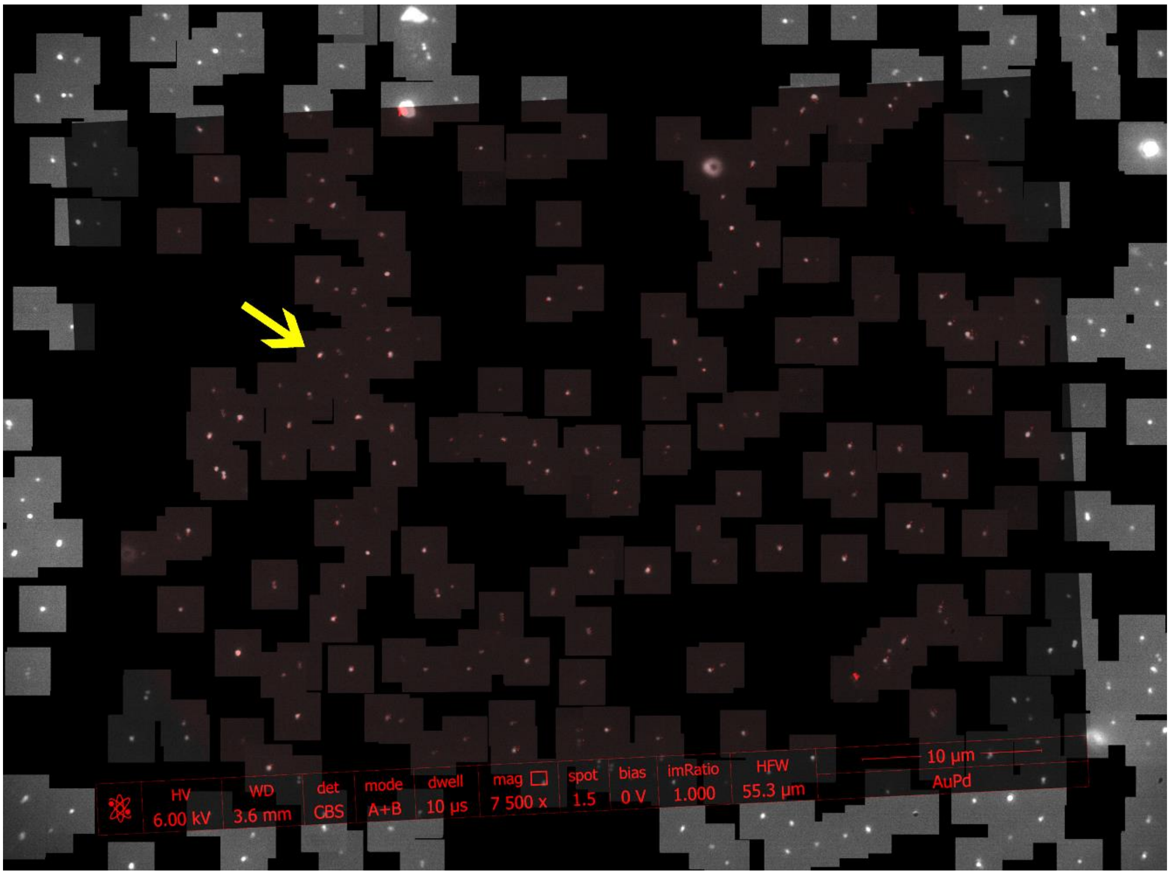

Supplementary figure 2. Overlayed SEM and optical microscopes images for the second view. The SEM image is highlighted red. The yellow arrow indicates the position of the event shown in Figure 7 of the article.

Resolution check by event simulation

The general idea of the simulation procedure is to simulate high-resolution (in terms of number of pixels) images and then downscale them to get the same pixel resolution as microscope's camera, thus, introducing the effect of sensor pixels size. Finally, we add the same level of random noise and process the images with the super-resolution method described in the article.

Measured PSF expansion

The measured optical microscope PSF for each polarization was 32×-upscaled by the bicubic interpolation method, thus, increasing the image resolution from 31×31 pixels to 992×992 and decreasing the effective pixel size from 27.5 nm to 0.86 nm.

Camera sensor noise estimation

The sensor noise level was measured by using empty image areas by estimating the mean value and the rms for each pixel. The mean pixel value was estimated to be 20.5 while the mean rms due to noise is 0.63. These values were applied to all generated images.

Resolution check by system response to a point-like source

In order to simulate the system's response to a point-like modulated source we generated a pixel with random parameters a, b and φ (see Eq. 5 in the article) at a random position inside a 1312×1312 empty image. Then, using the equation 5 of the article we generate a set of 8 greyscale images, each corresponding to the known experimental polarization angles. Then, each image is convoluted with the extended PSF of the corresponding polarization angle to get the diffraction-limited high-resolution image. After that we 32×-downscale images to get the resolution 41×41 pixels. Then, the image background is adjusted to be 20.5 and the gaussian pixel noise with the rms equal to 0.63 is introduced. Thus, we get a set of 8 images equivalent to those acquired by the optical microscope. Then we apply the same reconstruction procedure as described in the article to produce a 123×123 pixels super-resolution image. Repeating the above simulation for 100 events and averaging the value channel of the resulting images, we estimate the system's response averaged by all polarization angles, shown in Supplementary figure 3.

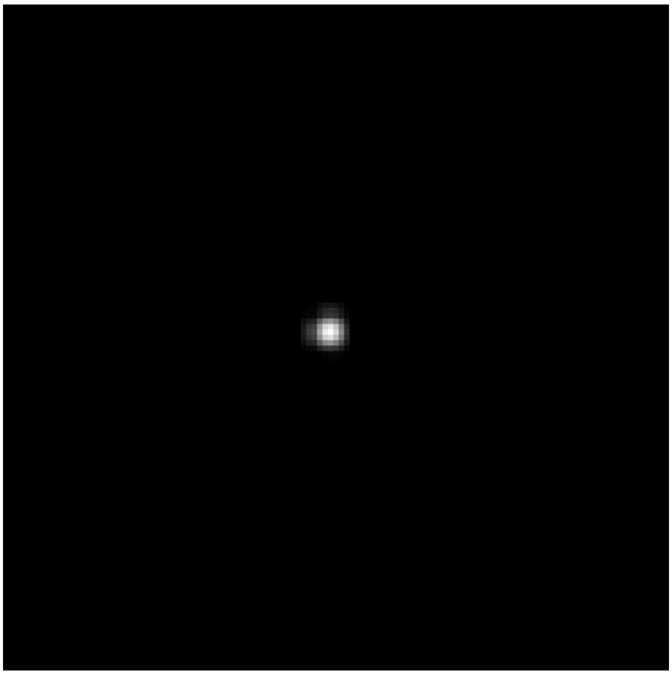

Supplementary figure 3. System's response to a point-like source. Image size 123×123 pixels. Pixel size 9.17×9.13 nm.

This image can be interpreted as the system PSF and, therefore, the resolution can be estimated as 2.9 σ_x, where σ_x = 1.7 ± 0.5 pixels, as measured from the gaussian fit of the PSF profile shown in the left panel of Supplementary figure 4. Therefore, also multiplying by the pixel size, one gets the resolution as 45 ± 12 nm.

This simulation also allows to estimate the accuracy of pixel phase reconstruction by plotting the difference between the reconstructed phase and the generated one as shown in the right panel of Supplementary figure 4. The pixel phase reconstruction

accuracy is estimated to be 36 ± 4 mrad. The pixel phase can potentially be used to further improve the resolution by distinguishing objects falling into adjacent pixels.

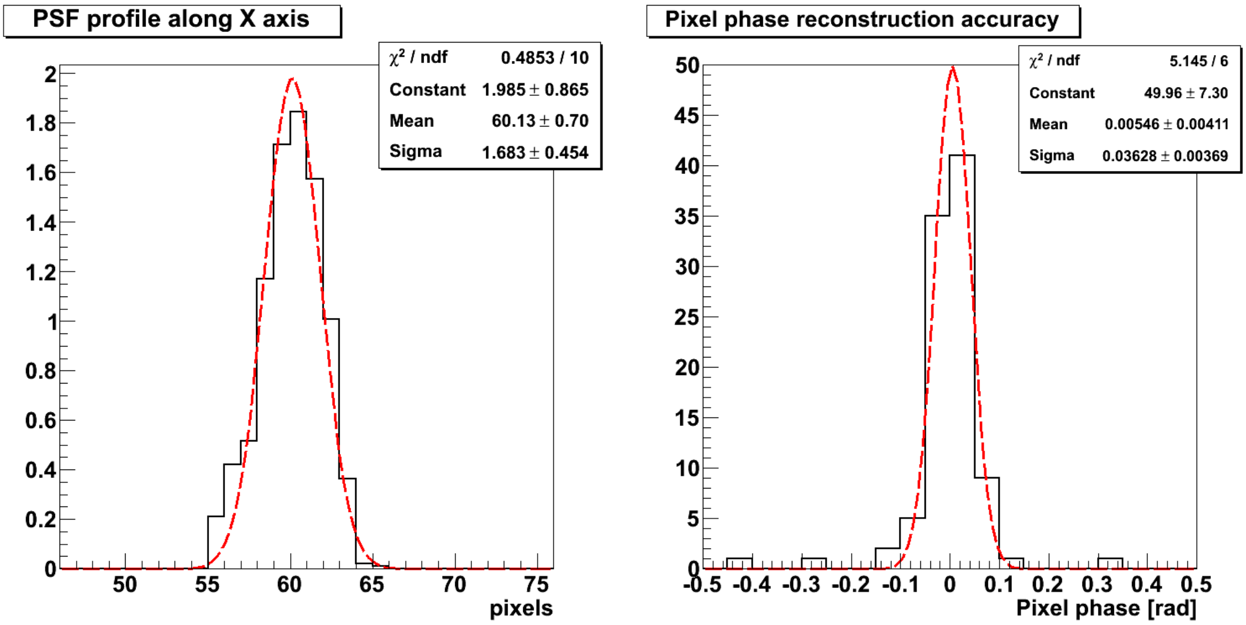

Supplementary figure 4. (left) System's PSF profile along the X axis. Pixel size = 9.173 nm. (right) Pixel phase reconstruction accuracy.

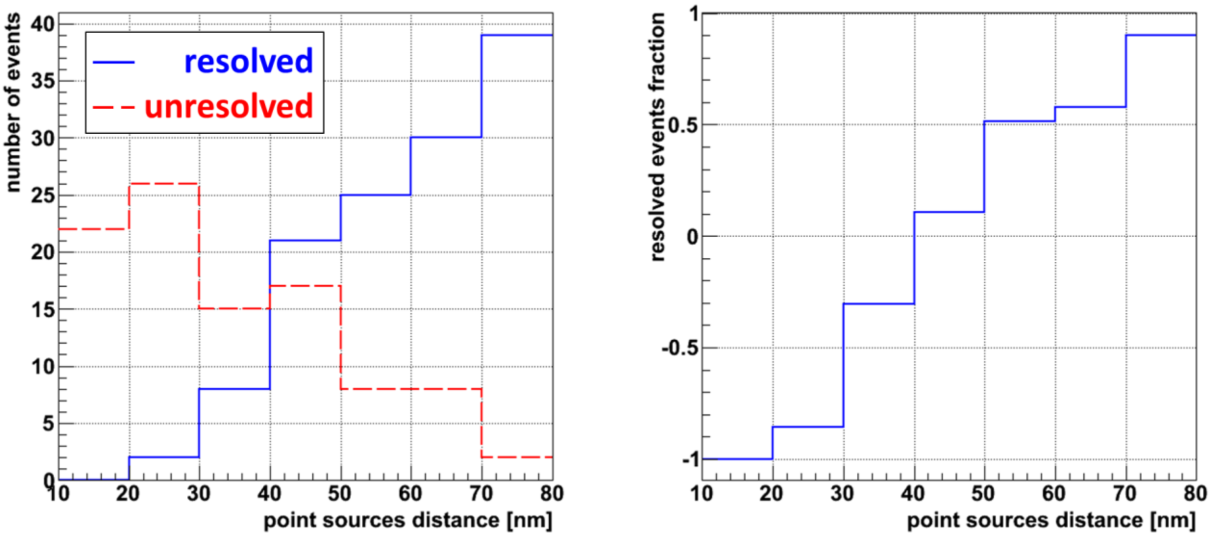

Supplementary figure 5. (left) number of resolved (blue solid line) and unresolved (red dashed line) events. (right) Resolved events fraction

Resolution check by simulating two close point-like sources

Another approach is to generate two nearby point-like sources and check the distance at which the system starts to resolve them. The generation procedure is the same as described above with the only difference that two pixels, instead of one, are generated with random parameters and at random positions inside an empty image. Then the images are convoluted with the extended PSF and are analyzed by the method described in the article to get super-resolution images.

Then, from each super-resolution image we extract the value channel and analyze it for the presence of two resolved peaks. The number of resolved and unresolved events is shown in the left panel of Supplementary figure 5 while the right panel shows the resolved events fraction defined as $F_{resolved} = \frac{N_{resolved} - N_{unresolved}}{N_{resolved} + N_{unresolved}}$. From these plots one can conclude that resolved events dominate for source distances greater than 50 nm which can be used as a measure of the method's resolution and is compatible with the resolution estimation of the previous section. The resolution gain in comparison with deconvolution methods is due to the additional information provided by the polarization modulation.

Resolution check by simulating grid-like structures

To study method's performance on grid-like structures, we carried out the simulation of a linear grid consisting of three close square nodes, shown in Supplementary figure 6a. The nodes have dimension of 25 nm and are spaced by 25 nm distance. The modulation phase of the central node if shifted by 90° with respect to the lateral ones (shown as different node colors). This modulation phase offset corresponds to the situation when the minimal brightness of the central node occurs when the lateral nodes have maximal brightness. Thus, the virtual absence of the central node allows resolution of the lateral ones. The reconstructed super-resolution image is shown in Supplementary figure 6d, and all nodes were reconstructed correctly along with their modulation phases confirming that the resolution of 50 nm is achievable in case of a linear grid. However, it needs to be noted that this resolution gain depends on the modulation phase difference of the two adjacent nodes: it is expected to be the factor of two for the modulation phase offset of 90°, whereas no gain is expected for zero offset. In case of intermediate offsets, the real shape of nodes as well as their positions are distorted but it is still possible to infer the presence of two or more objects by analyzing the reconstructed modulation phase (which appears as different colors in reconstructed images). However, a more elaborate procedure is required to reconstruct shapes and positions.

The proposed method works best for linear structures, like the reconstruction of charged particle tracks in NIT emulsion, the task for which the method was developed. For two-dimensional grids, like shown in Supplementary figure 7a, the resolution becomes worse due to higher probability for two neighboring nodes to have same or very close modulation phases. We have performed a simulation of 100 3×3 grid images, giving a random modulation phase to each square node. After the reconstruction, the volume channel was extracted from each resulting super-resolution image. Their superposition is shown in Supplementary figure 7b confirming that resolution of 100 nm is reachable in bidimensional case.

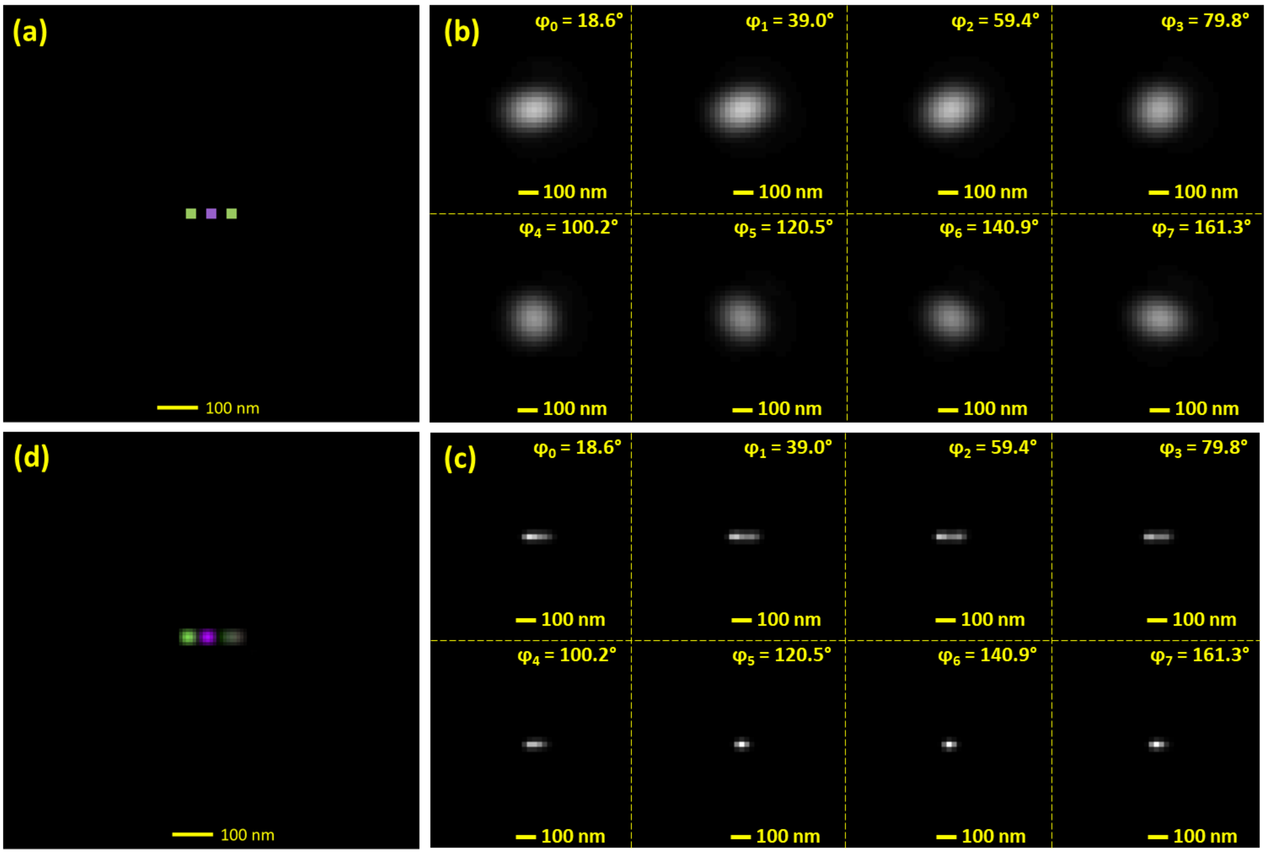

Supplementary figure 6. (a) Image of the modulated linear grid (pixel size 0.86 nm). Grid node size 25×25 nm, node-to-node distance 25 nm. Green and violet colors correspond to 45° and 135° polarization angles, respectively. (b) Convolution of the grid image with the measured PSF at indicated polarization angles (pixel size 27.5 nm). (c) Deconvoluted images (pixel size 27.5 nm). (d) Reconstructed super-resolution image (pixel size 9.17 nm).

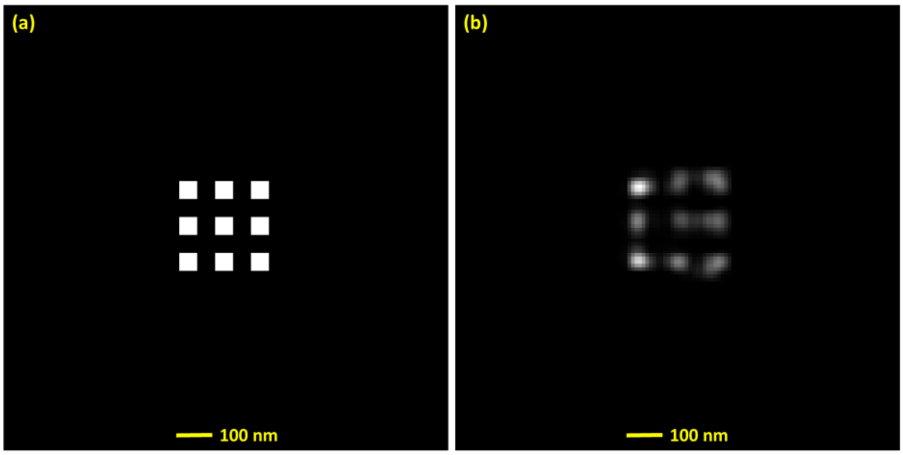

Supplementary figure 7. (a) Image of the 2D grid (pixel size 0.86 nm). Grid node size 50×50 nm, node-to-node distance 50 nm (b) Average of 100 reconstructed super-resolution images (pixel size 9.17 nm).
